# Transcriptome assembly and microarray construction for *Enchytraeus crypticus*, a model oligochaete to assess stress response mechanisms derived from soil conditions

**DOI:** 10.1186/1471-2164-15-302

**Published:** 2014-04-23

**Authors:** Marta P Castro-Ferreira, Tjalf E de Boer, John K Colbourne, Riet Vooijs, Cornelis AM van Gestel, Nico M van Straalen, Amadeu MVM Soares, Mónica JB Amorim, Dick Roelofs

**Affiliations:** 1 Department of Ecological Science, Faculty of Earth and Life Sciences, VU University Amsterdam, De Boelelaan 1085, 1081 HV Amsterdam, The Netherlands; 2 Department of Biology and CESAM, University of Aveiro, Campus Universitário de Santiago, 3810-193 Aveiro, Portugal; 3 The Center for Genomics and Bioinformatics, Indiana University, 1001 E. 3rd St., 47405-7005 Bloomington, IN, USA; 4 Current Address: Environmental Genomics Group, School of Biosciences, University of Birmingham, Edgbaston, B15 2TT Birmingham, UK

**Keywords:** Ecotoxicogenomics, Next-generation pyrosequencing, Invertebrate, Zinc, Annelid, 454 sequencing

## Abstract

**Background:**

The soil worm *Enchytraeus crypticus* (Oligochaeta) is an ecotoxicology model species that, until now, was without genome or transcriptome sequence information. The present research aims at studying the transcriptome of *Enchytraeus crypticus*, sampled from multiple test conditions, and the construction of a high-density microarray for functional genomic studies.

**Results:**

Over 1.5 million cDNA sequence reads were obtained representing 645 million nucleotides. After assembly, 27,296 contigs and 87,686 singletons were obtained, from which 44% and 25% are annotated as protein-coding genes, respectively, sharing homology with other animal proteomes. Concerning assembly quality, 84% of the contig sequences contain an open reading frame with a start codon while *E. crypticus* homologs were identified for 92% of the core eukaryotic genes. Moreover, 65% and 77% of the singletons and contigs without known homologs, respectively, were shown to be transcribed in an independent microarray experiment. An Agilent 180 K microarray platform was designed and validated by hybridizing cDNA from 4 day zinc- exposed *E. crypticus* to the concentration corresponding to 50% reduction in reproduction after three weeks (EC50). Overall, 70% of all probes signaled expression above background levels (mean signal + 1x standard deviation). More specifically, the probes derived from contigs showed a wider range of average intensities when compared to probes derived from singletons. In total, 522 significantly differentially regulated transcripts were identified upon zinc exposure. Several significantly regulated genes exerted predicted functions (e.g. zinc efflux, zinc transport) associated with zinc stress. Unexpectedly, the microarray data suggest that zinc exposure alters retro transposon activity in the *E. crypticus* genome.

**Conclusion:**

An initial investigation of the *E. crypticus* transcriptome including an associated microarray platform for future studies proves to be a valuable resource to investigate functional genomics mechanisms of toxicity in soil environments and to annotate a potentially large number of lineage specific genes that are responsive to environmental stress conditions.

## Background

The ecologically relevant soil-living worm *Enchytraeus crypticus* (Annelida; Oligochaeta; Enchytraeidae) has been widely used as model species in soil ecotoxicology for over two decades, both under International Organization for Standardization (ISO) and Organization for Economic Co-operation and Development (OECD) guidelines [1-4]. Ecotoxicological tests generally focus on survival and reproduction endpoints, i.e. organism effects, whereas in ecotoxicogenomics, responses at the transcriptome level are studied aiming to reconstitute the mechanisms preceding effects at organismal and/or population level (e.g. reproduction). In-depth information about the molecular mechanisms is provided allowing a better understanding of molecular pathways activated by toxicity, which will not be possible when using the classical tests alone [5].

Previously, transcript information with classical Sanger sequencing was generated for the test species *Enchytraeus albidus*[6] and has been very useful to further explore the response mechanisms of enchytraeids to various stressors, such as pesticides [7], heavy metals, nanoparticles [8], and soil properties [9]. However, the drawback of the current *E. albidus* microarray is that it contains a low number of transcripts, so a full transcriptomic assessment of stress response cannot be executed for this model. Thanks to the progress in sequencing technology, transcriptome and genome information can now be obtained for any non-genomic model species [10]. Transcriptome sequencing is of special interest, since it is directly targeted to obtain sequence information from transcribed regions in the genome [11,12]. To pursue a comprehensive transcriptome of an enchytraeid test species we selected *E. crypticus* instead of the more widely used *E. albidus*, despite the seemingly lower sensitivity to toxicants [3] of this species. Several advantages over the use of *E. albidus* have been recently identified, such as easy culturing conditions in agar media, higher reproductive rate, lack of cryptic speciation, and a shorter reproduction cycle (3 weeks instead of 6) [3].

Given the clear advantages of using *E. crypticus* for soil quality assessment, the study presented here aims at studying its transcriptome by *de novo* transcriptome pyrosequencing and annotation. A previous transcriptome study showed that it is beneficial to pool RNA from multiple treatments in order to obtain a wider range of expressed transcript sequences [13]. Thus, we decided to set up a similar approach to get transcriptome-wide information from *E. crypticus* and sampled mRNA from multiple exposure conditions, including chemical [3], physicochemical (moisture, temperature, pH) and developmental stages. The quality of the assembled transcriptome was critically assessed according to the guideline provided by Gibson et al. [14] by evaluating sequence quality, assembly quality, overall transcriptome completeness, and transcriptional activity. Subsequently, a high-density microarray platform was constructed and validated using zinc-exposed organisms. This platform will be a valuable resource to conduct gene expression studies in future ecotoxicogenomic research.

## Methods

### Test organism culturing conditions and experiment overview

The annelid *Enchytraeus crypticus* (Oligochaeta: Enchytraeidae), commonly known as potworm, has been cultured for several years in laboratory at the VU University Amsterdam. Cultures are kept in agar substrate prepared with aqueous soil extract, fed ad libitum with ground oatmeal, at 16°C with 75% relative humidity and 16/8 h light/dark photoperiod [1,3,4]. For this study we selected, for each biological replicate per exposure, 5–20 *Enchytraeus crypticus* adults with developed clitella, similar size, and an average fresh weight of 1.4 (±0.2) mg per adult. In total, 40 different experimental conditions were included: 12 chemical exposures 12 temperature treatments, 5 soil moisture treatments, 3 pH treatments, 4 different developmental stages, plus associated control conditions (Additional file 1).

### Test soils

The reference natural LUFA 2.2 soil (Speyer, Germany) was used in all tests, except for the pH treatments, which were performed using OECD artificial soil [1,4].

LUFA 2.2 soil is a loamy sand soil sieved at 2 mm with pH (CaCl_2_) 5.5, cation exchange capacity (CEC) 10 meq/100 g, maximum water-holding capacity (WHC_max_) of 46%, organic matter content of 4%, grain size distribution of 6% clay, 14% silt and 80% sand. For testing, control moisture was adjusted to 50% of the WHC_max_.

The OECD artificial soil was prepared according to the standard procedure [1] and further used to test various pHs adjusting it by adding predetermined amounts of calcium carbonate (CaCO_3_).

### Treatments

Toxicity tests were performed following the standard guidelines [1,4]. Chemical test concentrations were based on previous results [3] from which the 3-week reproduction EC50 was estimated. This EC50 was used as a reference exposure concentration in the short-term (48 h) experiments from which the mRNA pool was derived. Test chemicals (Additional file 2) included cadmium chloride hemipentahydrate (Cd), carbendazim (Car), phenanthrene (Phe), pentachloroaniline (PCA) and 3,5-dichloroaniline (DCA). Chemical formulas, chemical abstract service (CAS) numbers and concentrations are described in Additional file 2. Non-water soluble chemicals were solubilized in acetone. For the acetone control and acetone chemical spiking the following was performed: 25 gr of dry LUFA 2.2 soil was spiked with acetone (10 mL; CAS No. 67-64-1; purity ≥ 99.8%; supplied by Sigma Aldrich) or the appropriate organic-acetone solution for Phe, Car, PCA and DCA, equilibrated for 24 h, left to evaporate overnight, mixed with dry soil (75 g) and homogeneously moistened to 50% of the WHC_max_. The soil batches were used immediately after being prepared. For the metal exposures (Additional file 1), dry LUFA 2.2 soil (100 g) was spiked with the specific metal-aqueous solution (10 mL) to reach the nominal concentration and 50% of the WHCmax in the soil batch. The soil was kept under standard conditions while equilibrating for 21 days prior to exposure. Two replicates were prepared per test condition (Additional file 1).

Per replicate, twenty adult *E. crypticus* were introduced into each glass vial (100 mL) containing 30 g of soil prepared as described above. Food (2 mg) was supplied and vessels were closed with perforated aluminum foil. The exposures lasted 48 h at 20°C, with 75% relative humidity and 16/8 h light/dark photoperiod. After 48 h of exposure, the individuals in each replicate were sampled, carefully rinsed in deionised water, transferred to a cryotube, snap-frozen with liquid nitrogen and stored at -80°C.

Zinc (Zn) exposures were performed in order to validate the microarray platform. The EC50 for inhibition of reproduction was used (De Boer et al., unpublished data). For the microarray analysis, *E. crypticus* adults were exposed to 145 mg Zn/kg soil (EC50) and control soil for 4 days using four replicates, containing 5–7 individuals per replicate.

Temperature, moisture and pH treatments were performed according to the guidelines, with standard test conditions as described in the previous paragraph, except for the specific factor being studied (Additional file 1). The temperature exposures lasted 0.5, 24, 48 or 96 h, at the temperatures -10, 0, 10, 20, 30 and 40°C. The moisture exposures lasted 24, 48 or 96 h, using soils moistened to 20, 50 and 90% of WHC_max_. The pH exposures lasted 48 h, using soils with pH 3, 4.5 and 6.

*Enchytraeus crypticus* samples in distinct developmental stages were taken from synchronization of cultures, which were obtained by transferring thirty adults with developed clitella to fresh culture boxes that were kept at identical culturing conditions as the source culture. Every 6 hours, cocoons were collected (20 and 50 cocoons per 6 hour time-point), immediately snap-frozen with liquid nitrogen and stored at -80°C. Twenty synchronized cocoons were carefully transferred into fresh culture media and kept at 20°C. After twenty days twenty juveniles were collected into a cryotube, immediately snap-frozen with liquid nitrogen and stored at -80°C.

### RNA isolation, RNA pool and cDNA library construction

Total RNA was collected from the 90 individual samples of *Enchytraeus crypticus* as listed in Additional file 1. All RNA extractions were performed using the SV Total RNA Isolation System (Promega) following manufacturer’s protocol. All samples were homogenized with liquid nitrogen in the cryotube before RNA extraction. Addition of sterilized quartz sand to the liquid nitrogen was needed to disrupt and homogenize cocoons sufficiently.

**Figure 1:**
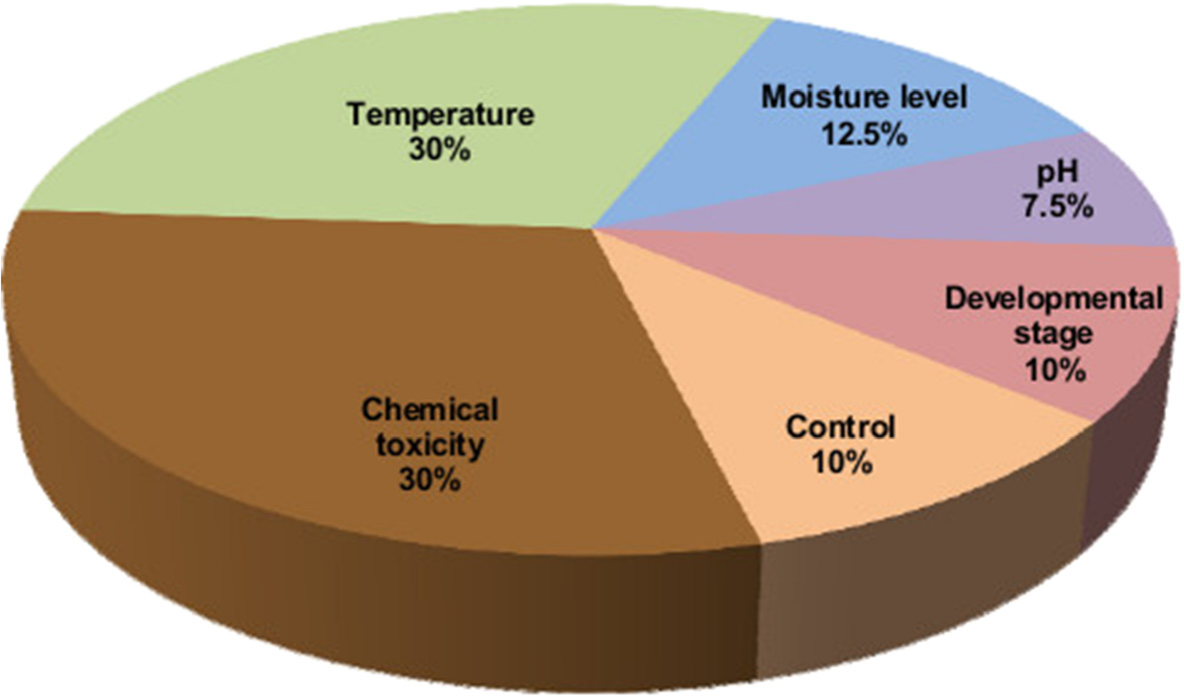
**Composition of the*E. crypticus*RNA pool used for transcriptome sequencing, showing RNA masses (%) from distinct test conditions.** This RNA pool included balanced RNA masses (%) from 40 distinct exposure conditions: twelve chemicals, twelve temperature treatments, five for soil moisture treatments, three pHs, four developmental stages, and four control conditions.

The overall quality of RNA samples was evaluated in terms of purity and integrity of RNA by means of a NanoDrop ND-1000 UV–VIS spectrometer (Thermo Fisher Scientific) and agarose gel electrophoresis. RNA sample quality was verified regarding high RNA concentration, absorbance ratios A260/A280 in the range 2.0 - 2.2, and A260/A230 above 1.8. Samples with lower absorbance ratio were ethanol-precipitated in order to improve the quality. Equivalent amounts of RNA mass per test condition were pooled together, with a total of 10 μg RNA from all samples of *E. crypticus.* Figure 1 illustrates the proportion of RNA input per test condition represented in the pool that was used for sequencing. Normalized 454-sequencing libraries were constructed from an equal-molar pool of RNA obtained from the unique exposure samples described above using the procedures described in Meyer et al. [15]. After the final purification step the library was stored at -20°C until sequencing.

### Transcriptome sequencing and assembly

Cytochrome oxidase I and 18S rRNA gene sequencing of 10 individuals yielded only one polymorphic site (Roelofs et al., unpublished data), providing strong evidence that this strain is highly inbred. Thus, the assembly procedure is not hampered by the presence of genetic variation within the cDNA library. This library was submitted to one full-plate sequencing run using a 454 Roche GS FLX pyrosequencing instrument using Titanium chemistry (454 Life Sciences Corporation), following manufacturer’s protocol and methods previously described [16]. After 454 sequencing, the generated sequence reads were assembled using Newbler (software v. 2.5.3 from 454 Life Sciences Corporation) in *de novo* mode and default parameters.

### Transcriptome annotation

Transcriptome annotation was performed through the ISGA transcriptome analysis pipeline, available from the Center for Genomics and Bioinformatics at Indiana University (http://isga.cgb.indiana.edu/Pipeline/View?pipeline=248). First, sequence homology to known metazoan proteins was obtained by submitting contigs and singletons to BLASTx searches against NCBI’s non-redundant database and dbEST [17]. Moreover, protein domains were identified among the 6 frame translations of the assembled sequences using Pfam, TIGRfam and HMMER3 searches. Open reading frames were determined with the ORFpredictor software on the proteomics server of Youngstown state university [18]. Orthology and paralogy was assigned by BLASTx against orthoMCL databases [19]. Assembly quality was tested by retrieving BLASTx hits against the *Drosophila* orthologs in the CEGMA core eukaryotic genes dataset [20]. Transcriptome quality assessment was performed according to the guidelines described by Gibson et al. [14]. In short, open reading frame (ORF) lengths, EST nucleotide lengths and GC contents of annotated and unannoted ESTs were compared using a Welch’s *t*-test in R version 2.15. Furthermore, transcript abundance (microarray intensity) was compared between contigs and singletons using Welch’s *t*-test. Finally, the interactive pathway explorer iPATH was used to identify metabolic, regulatory and biosynthesis pathways based on enzyme code (EC) numbers [21].

### Probe design for microarray construction

Oligonucleotide probes were designed according to the thermodynamics/technical features of gene expression microarrays within the eArray webtool of Agilent (Agilent Technologies). The workflow is summarized into these consecutive steps: the target sequences were selected by length threshold ≥300 bases, converted into sense orientation, and then submitted to eArray for probe design. In order to avoid cross-hybridization, the probes that could cross-hybridize with any sequence other than the specific target (indicated by a probe sequence similarity of >90%), were discarded. A final microarray design was created from the probe group (86,412 probes) that were printed in duplicate (one probe per contig/singleton represented twice) onto a 4 X188 K platform. The array design was supplemented with 4,854 additional Agilent control probes (random probes for background correction plus spike-in positive control probes), so that in total 177,678 probes were present.

### Microarray validation and data analysis

RNA samples were extracted from control and EC50 Zn-exposed *E. crypticus* adults. The RNA was then precipitated with ethanol to remove any Guanidine Thiocyanate traces from the SV RNA extraction procedure and to increase its concentration. RNA concentration and quality was determined on the Nanodrop nd1000 spectrophotometer (Thermo Scientific) and Bioanalyzer 2100 (Agilent Technologies). RNA samples were labeled using the Low Input Quick Amp kit (Agilent Technologies) according to the manufacturer’s instructions and as described [22]. Four replicates were labeled using Cy-3/Cy-5 dye swaps. Labeled samples were hybridized to the microarray as described above. Microarrays were scanned using an Agilent microarray scanner and raw data was extracted with Feature Extraction software version 10.7.3 (Agilent Technologies). Microarray raw intensities were LOESS normalized with a background subtraction according to the Edwards method which contained a minimal offset of 30 [23,24]. Significant differential expression of genes was determined by directly contrasting the control signal to the EC50 exposure samples and fitting a linear model through the data all using the Limma package for R/Bioconductor [25] after which pP-values were adjusted for multiple testing according to the Benjamini-Hochberg method. A Gene Set Enrichment analysis was performed in Blast2GO using a Fisher’s exact test again using a p-value correction with the false discovery rate (FDR) method by Benjamini-Hochberg [26].

## Results and discussion

### Transcriptome sequencing and assembly

Quality filtering of the reads was performed before assembly by applying default parameters for nontrace sequences as described by Vera et al. [11]. After the pyrosequencing run, 68% (1,496,497) of the total number of wells passed the quality control filter, representing 645,342,910 bases of raw NGS data. The reads obtained from this run had median and average lengths of 479 and 431 bases, respectively; and the longest read was 1,195 bases long (Additional file 3). The obtained large number of bases into long reads indicated high quality transcriptome-wide sequence information. The assembly was performed on all 1,478,792 quality-controlled reads summing up 96.0% of raw NGS data (619,758,522 bases), after trimming of adaptor sequences (Table 1). Newbler aligned 91.4% and assembled 86.1% of all reads, resulting in 27,296 contigs and 87,686 singletons (Table 1). The median coverage was 10, average was 15 and maximum coverage was 190.

**Table 1:** **Sequencing results and assembly statistics (using Newbler) for** ***E. crypticus*** **transcriptome sequences**

**Sequencing results**	**Total**	**Aligned**	**Assembled**
Number of reads^1^	1,478,792	1,351,016	1,273,500
Number of bases	619,758,522	569,108,879	663,968,932
**Assembly statistics**	**Singletons**	**Contigs**	**Isotigs**
Number of sequences	87,686	27,296	24,748
Number of bases	34,418,269	28,992,517	31,886,676
Average sequence size (bp)	392.	1,062	1,288
Length of N50 sequence (bp)^2^	475	1,354	1,443
Largest sequence size (bp)	1,195	6,693	7,689

^1^After quality filtering steps and removal of outliers such as adaptor sequences and repeats.

^2^N50 is a weighted median statistic, such that 50% of all bases are contained in sequences not inferior to N50 length.

In the present study the largest singleton was 1,195 bp long. The largest contig was 6,693 bp long and 13,063 contigs were over 1,000 bp long. N50 is a weighted median statistic, such that 50% of all bases are contained in sequences not inferior than the N50 length. The N50 for contig length was 1,354 bp. The assembly procedure using Newbler considers alternative splicing, and this resulted in the integration of 27,296 contigs into 24,748 isotigs representing candidate transcripts. Furthermore, the assembler indicated that the isotigs are represented by 21,721 isogroups, defined as candidate gene sequences. The N50 for isotigs was 1,443 bp and the largest isotig was 7,689 bp long. The largest contig count was 13 per single isotig and 28 contigs per single isogroup. Moreover 80% of isotigs and 90% of isogroups comprised one single contig.

### Quality control of transcriptome completeness

**Figure 2:**
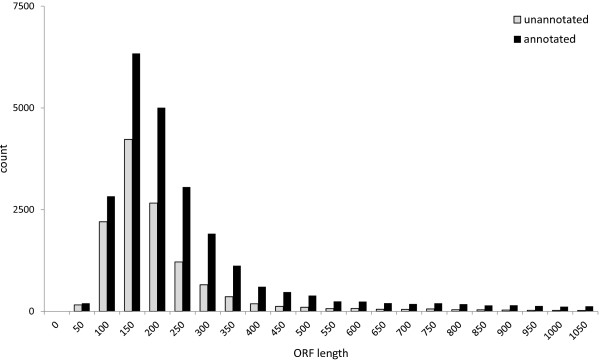
**Distribution of the nucleotide lengths of open reading frames for annotated and unannotated contigs.** Annotated contigs are shown in black and unannotated contigs in grey. Open reading frame lengths were predicted with ORFpredictor.

Several quality control analyses were performed in order to assess quality and completeness of the present transcriptome. The Core Eukaryotic Genes Mapping Approach (CEGMA) contains a core set of 458 single copy genes present in all eukaryotic genomes that can be used to assess the completeness of genome and transcriptome assemblies. The *Drosophila melanogaster* reference set of Core Eukaryotic Genes (CEGs) [20] was used in BLASTx searches against the *E. crypticus* transcriptome. At a threshold expect value of 10^-25^, 395 of the 458 CEGs could be retrieved, indicating that the transcriptome is about 86% complete (Additional file 4). This percentage increased slightly to 92% when we used a more relaxed e-value threshold of 10^-10^. We also asked the question whether the lack of annotation (see below) is due to technical errors of the sequencing run or assembly errors. Therefore, we verified whether the annotation process was influenced by the quality of the sequence data. To that end, we compared open reading frame (ORF) length, mean sequence length, percentage of start codons and mean GC content of contigs and singletons according to the quality control methods and guidelines for transcriptome sequence data described by Gibson et al. [14]. Table 2 summarizes the outcome of this analysis. Firstly, annotated sequences were on average significantly longer than unannotated (Welch test, t = 5.6567, df = 77608.06, p-value = 1.5 e-08). Likewise, sequence length of annotated ORFs (as predicted by ORFpredictor) was significantly longer than unannotated ORFs (t = 83.1603, df = 42680.87, p-value < 2.2e-16, Figure 2). Moreover, ORFpredictor predicted a higher percentage of start codons in annotated sequences when compared to unannotated: as much as 84% of all contigs contained a start codon. GC content was significantly higher in annotated sequences when compared to unannotated (t = 160.981, df = 80870.32, p-value < 2.2e-16). A study comparing *Anopheles’* and *Drosophila’s* genomes with their corresponding proteomes showed that orphan (unannotated) sequences are indeed protein-coding, and mostly code for shorter proteins when compared to currently annotated proteins [27]. Therefore, unannotated ORFs are expected to be more abundant among shorter predicted ORFs as summarized in Table 2 and especially in relation to contigs (Figure 2).

**Table 2:** Comparison of annotation success between contigs and singletons

	**Annotated**	**Unannotated**
Mean ORF length		
Contigs	349	197
Singletons	226	145
Mean length		
Contigs	1,324	874
Singletons	447	393
Containing start codon (%)		
Contigs	84.8	68.5
Singletons	67.7	49.8
Mean GC-content (%)		
Contigs	43.4	37.9
Singletons	46.9	38.6

### Functional annotation of the *E. crypticus* transcriptome

As this represents the first *E. crypticus* transcriptome sequencing study, it was crucial to include all sequence data in the analyses, to avoid losing a priori valuable biological information. Table 3 summarizes the functional annotation of *E. crypticus’* transcriptome (we refer to Additional file 5 for details of annotations for each contig and singleton). The annotation process retrieved information on Gene Ontology (GO) terms, functional protein domains via InterProScan [28], enzyme codes and enzymatic pathway information from Kyoto Encyclopedia of Genes and Genomes (KEGG). From the total number of singletons and contigs, a BLASTx match was obtained for 29% and 50% respectively (Table 3). This implies that between 50% (contigs) and 70% (singletons) of the sequences do not show homology to any other sequence present in the investigated databases. Yet, over 77% of unknown contigs and 65% of unknown singletons are transcribed as determined by microarray analysis (see section on microarray results), suggesting that they may represent protein-coding genes as well as non-coding trasncripts such as microRNAs and long non-coding RNAs.

**Table 3:** **Summary of** ***E. crypticus*** **transcriptome annotation**

		**Contigs**		**Singletons**	
No. sequences	Total	27296		87686	
	BLASTx match	13,745	(50%)	25,347	(29%)
	With GO terms	12,133	(44%)	21,890	(25%)
	With mapped GO terms	9,401	(34%)	16,619	(19%)
	With enzyme codes	2,525	(9%)	4,253	(5%)
No. GO terms for…	Biological processes	24,577		42,989	
	Molecular functions	17,484		30,356	
	Cellular components	12,996		20,726	
No. Orthologs	orthoMCL	4,875		11,278	

**Figure 3:**
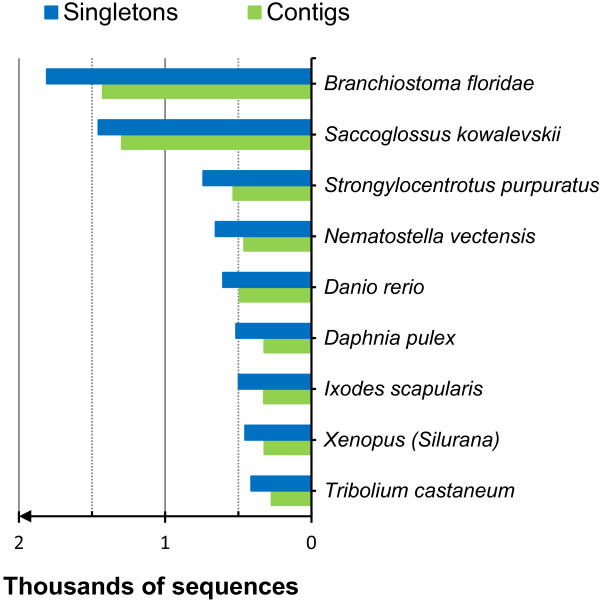
**Distribution of BLASTx top-hit species for*E. crypticus*singletons and contigs.** The BLASTx top-hit species for a given sequence is the best-hit for a given BLASTx result among all Blast results. The best alignment is the one with the highest sequence similarity and the lowest e-value. Blue represents BLASTx hits using singletons; green represents BLASTx hits using contigs.

Gene Ontology terms were assigned to 25% of singletons and 44% of contigs; overall more than 34,000 sequences were mapped. In total 26,020 sequences (19% of total singletons and 34% of contigs) received a full annotation (Table 3). Figure 3 shows the BLASTx top-hits for both singletons and contigs, listing the top species for which highest sequence similarity was obtained against *E. crypticus* sequences. OrthoMCL analysis [29] revealed that 4,875 contigs show orthology to one or more organisms in the orthoMCL database. Within singletons we revealed orthology for 11,278 sequences.

Most singletons and contigs showed high similarity with invertebrate deuterostomes such as: the chordate *Branchiostoma floridae* (lancelet, GenBank Taxonomy ID: 7739), the hemichordate acorn worm *Saccoglossus kowalevskii* (ID: 10224), and the purple sea urchin *Strongylocentrotus purpuratus* (ID: 7668) (Figure 3). BLASTx top-hits for 1,810 singletons and 1,428 contigs were obtained by comparison with *B. floridae*, while top-hits for 1,457 singletons and 1,298 contigs were retrieved by comparison with *S. kowalevskii*. The large number of matches between *E. crypticus* and chordates confirms the suggestion that the lophotrochozoan gene complement represents a rather complete bilaterian genome and that relatively few genes were lost in comparison to ecdysozoan genomes [30,31]. This was recently confirmed by investigating the syntheny in three spiralian genomes (molluscs and annelids). They showed that the analysed worm and snail genomes are more similar to invertebrate deuterostomes when considering macrosynteny (large chromosomal regions), microsynteny (a group of 3 or more genes whose orthologous are linked in two or more genomes) of 469 gene clusters including the Hox gene complex [31], despite the fact that molluscs and annelids are evolutionary more closely related to protostomes such as arthropods, nematodes and flatworms.

Additional file 6 illustrates the general distribution of GO terms across the directed acyclic graph (DAG) structure at level 4, which is based on the percentages of mapped sequences for singletons and contigs with a cutoff at 7%. In total, thirty GO terms represented the most abundant annotation of singletons and contigs. When contigs are compared to singletons, we observe that GO term distribution is balanced with regard to biological process as well as cellular compartment. In contrast, GO terms related to the molecular functions cation binding and nucleotide binding are clearly more present within contigs, while GO terms related to the molecular functions protein binding, small molecule binding, hydrolase activity, ion binding, transferase activity and nucleic acid binding are more present within singletons. This suggests that certain molecular functions may be associated with transcript length, but further research should provide more detailed information on this.

Finally, the enzyme numbers, retrieved from the annotation of contigs and singletons, were submitted to iPATH interactive pathway explorer to verify to what extent metabolic pathways and biosynthesis pathways are represented in the transcriptome. As shown in Additional file 7, the present *E. crypticus* transcriptome comprises most of all essential metabolic pathways. Metabolism for terpenoids is not represented as such pathways are exclusive to plants.

### Microarray validation and data analysis

**Figure 4:**
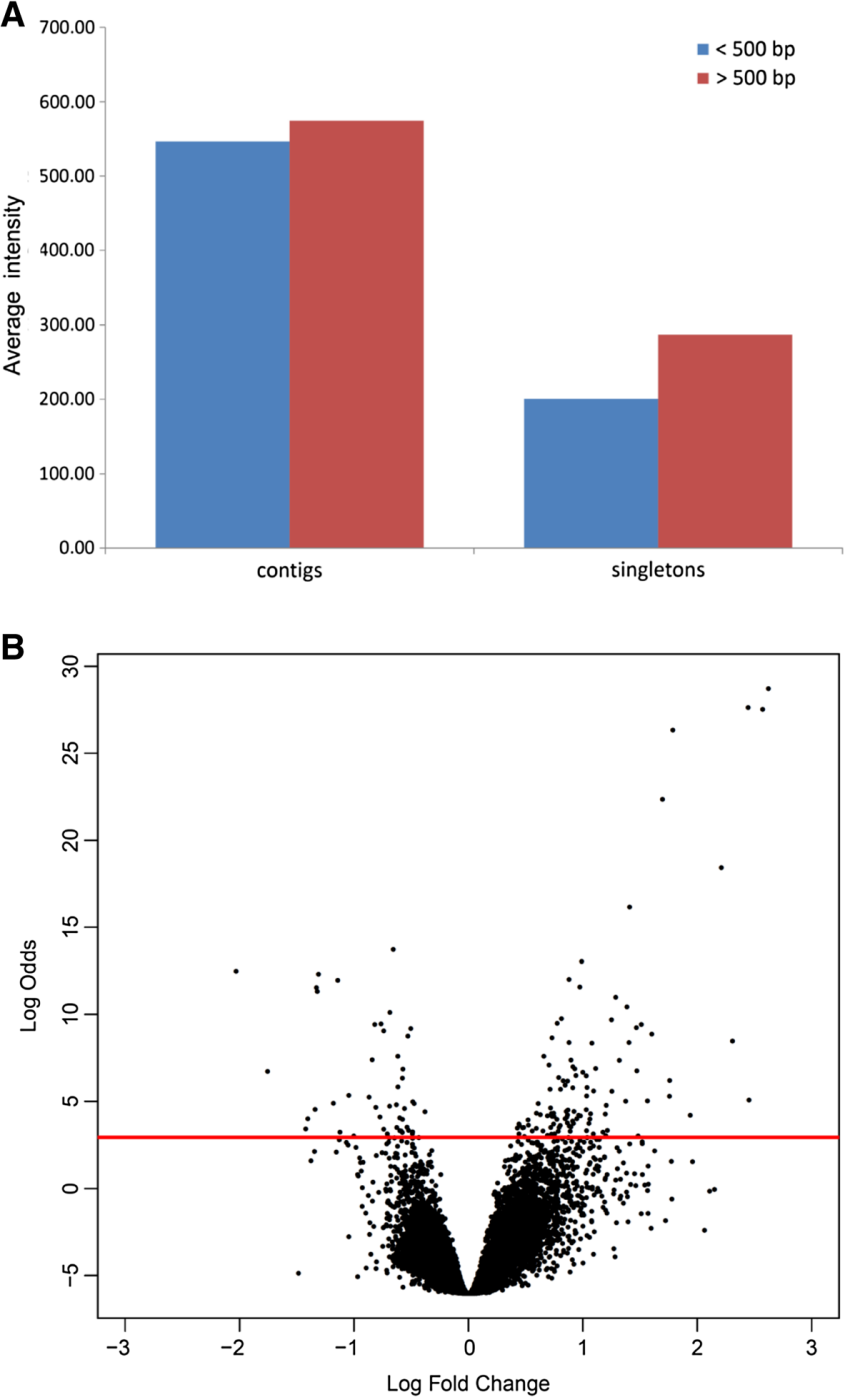
**Microarray hybridization data comparing zinc-exposed worms to control worms. A**: Contig intensities *vs* singleton intensities. Blue represents sequences below 500 bp, red represents sequences above 500 bp. **B**: volcano plot. X-axis, log2 differential expression ratio, Log odds of differential expression significance (Log(p)-Log(1-p)). Red line represents the Log odds ratio for FDR corrected p = 0.05.

The Agilent 4x180k microarray platform has a maximum capacity of 180,880 spots. After quality filtering of the contigs by eArray this turned out to be sufficient to include a probe for each contig and singleton in duplicate on the array. Sequence orientation was verified by ORF orientation upon translation, and showed that directional pyrosequencing was successful, because only about ~180 sequences were identified to be 3&vprime; to 5&vprime; orientated. We made sure that all annotated contigs and singletons were represented on the array. The *E. crypticus* array was tested in a gene expression profiling experiment, in which we studied differential expression in between control animals and animals exposed to the zinc EC50 on reproduction. This also allowed us to quantify the percentage of contigs and singletons that are actually transcribed in the control and in a stressful condition. Since the array contains 600 negative control spots (random synthetic 60 mer probes without known homology to biological DNA sequences), we quantified a mean background hybridization signal for the Cy-3 and Cy-5 channels separately by taking the average intensity of these spots per channel for all microarrays and calculating the overall average intensity for the red and the green channel. Subsequently, 1X the standard deviation (SD) of this average intensity (27.5 SD =2.86 for green channel and 118.14 SD = 6.86 for red channel) was used as a cut-off value to verify if a sequence was actually transcribed. This resulted in 67% of all probes showing transcriptional activity, which is comparable to previously reported percentage of detectable levels of condition-dependent transcription [14]. We calculated absolute average intensity values of all contigs and compared that to average absolute intensity values of all singletons (Figure 4A). The average intensity values for contigs was almost 3 fold higher than for singletons, indicating that the majority of probes derived from singletons show lower average signal intensity. Indeed, a *t*-test confirmed that significantly more contigs were detected when compared to singletons (one sample *t*-test, t = 32.3854, df = 63057, p-value < 2.2e-16, Figure 4A).

Unknown sequences may include intronic regions or untranscribed sequences that remained in the library despite DNAse I treatment followed by reverse transcription and cDNA synthesis. If this is the case, probes derived from such sequences do not represent functional genes and will not show a hybridization signal. Remarkably, 77% of probes derived from unknown contigs and 66% probes derived from unknown singletons showed detectable levels of transcription. This suggested that most unannotated sequences belong to genomic regions that are being transcribed.

Additional file 8 shows MA plots of all probes (A), contigs (B) and singletons (C). Log2 average intensity values range between 4.9 and 18, which is very well in line with average microarray hybridization data [32]. The fact that M is independent of A over a large range demonstrates sufficient linearity of the responses over a large dynamic range of microarrays signal intensity. Still, a substantial collection of probes seem to show very low average intensity values. As mentioned above, these low intensity probes were most abundant among singletons.

Exposure to zinc resulted in 522 significantly regulated transcripts (Benjamini-Hochberg adjusted p < 0.05), of which 210 are annotated (Additional file 9). Figure 4B represents the volcano plot of this experiment. Among these transcripts, two were annotated as zinc efflux proteins and are both up-regulated between 1.4 and 8.8 fold (contig19147 and singleton GYUW2K402JE6WU). Additionally, a zinc transporter 10 was identified (contig24978), which was 4.4 fold up-regulated. Also, a zinc cadmium resistance protein (singleton GYUW2K401AMILL) was 1.6 fold up-regulated. These up-regulated transcripts are associated with zinc stress, indicating that an active efflux mechanism is induced aiming to remove excess zinc ions [33,34].

In order to retrieve biological processes affected by zinc we performed GO enrichment analysis [26]. Table 4 lists the 21 GO terms that were significantly enriched among the significantly regulated and annotated transcripts. The relatedness of these terms is drawn in a cyclical graph in Additional file 10. The process of protein translation, assembly and localisation targeted to membranes is represented by 13 GO terms. It is tempting to speculate that zinc exposure adversely affects protein metabolism, but experimental evidence is needed to confirm this. Nevertheless, this is in agreement with the observation that zinc is an essential metal serving as a critical structural component of many zinc-finger proteins with diverse functions [35]. The affected process of targeted localisation to membranes is also in line with the observation that efflux transporters are up-regulated, because these proteins have to be targeted and assembled in the cellular membrane to function properly.

**Table 4:** GO term enrichment analysis from zinc exposure

**GO-ID**	**Term**	**Category**	**p FDR**^**1**^	**# Sig.**^**2**^	**# Ann.**^**3**^
GO:0006614	SRP-dependent cotranslational protein targeting to membrane	P	4.78E-05	8	64
GO:0006613	Cotranslational protein targeting to membrane	P	4.78E-05	8	67
GO:0045047	Protein targeting to ER	P	4.90E-05	8	74
GO:0072599	Establishment of protein localization in endoplasmic reticulum	P	4.90E-05	8	74
GO:0070972	Protein localization in endoplasmic reticulum	P	3.21E-04	8	99
GO:0006612	Protein targeting to membrane	P	4.38E-04	9	149
GO:0022625	Cytosolic large ribosomal subunit	C	2.05E-03	6	56
GO:0019058	Viral infectious cycle	P	2.32E-03	8	140
GO:0006415	Translational termination	P	2.32E-03	6	60
GO:0072594	Establishment of protein localization to organelle	P	2.73E-03	8	147
GO:0022415	Viral reproductive process	P	3.97E-03	8	157
GO:0000184	Nuclear-transcribed mRNA catabolic process, nonsense-mediated decay	P	1.22E-02	6	86
GO:0016032	Viral reproduction	P	1.49E-02	9	257
GO:0015934	Large ribosomal subunit	C	1.59E-02	6	93
GO:0019083	Viral transcription	P	1.85E-02	6	98
GO:0019080	Viral genome expression	P	1.85E-02	6	98
GO:0043624	Cellular protein complex disassembly	P	2.52E-02	6	105
GO:0000956	Nuclear-transcribed mRNA catabolic process, nonsense-mediated decay	P	2.75E-02	7	162
GO:0022626	Cytosolic ribosome	C	2.75E-02	6	109
GO:0043241	Protein complex disassembly	P	3.32E-02	6	114
GO:0006402	mRNA catabolic process	P	4.18E-02	7	177

^1^False discovery rate corrected significant p-value (derived from Fisher exact test); ^2^number of significantly regulated transcripts containing the particular GO ID; ^3^number of transcripts on the array containing the particular GO ID.

Unexpectedly, five GO terms representing biological processes associated with viral transcription and reproduction were significant in the enrichment analysis. Closer inspection of the transcripts from these GO terms showed that most of these genes are associated with transposon activity (e.g. up-regulated singleton transcript GYUW2K402IXZSO, RNA-directed DNA polymerase from mobile element jockey). Jockey is a Non-Long Terminal Repeat retrotransposon (N-LTRs), commonly found in invertebrates and amplifies via an RNA intermediate [36]. The autonomous retrotransposition mechanism of N-LTRs is facilitated by a single open reading frame containing polymerases with zinc-finger domains for nucleic acid binding [37]. Apparently, zinc exposure inactivates retrotransposition, since all these transcripts are down-regulated (Additional file 9). Our results suggest that excess zinc ions may impair proper nucleic acid binding facilitated by the zinc-finger domains of the N-LTR open reading frame. The final three processes among the GO-enriched terms (Table 4) are involved in RNA catabolism.

Finally, we compared the *E. crypticus* transcriptome to transcript sequences of the sister species *E. albidus* available in Genbank and EnchyBASE [6] by using BLASTx. A total of 2100 *E. albidus* sequences clustered into 1124 unique gene clusters, of which 856 showed highly significant BLASTx similarities to contigs in the *E. crypticus* transcriptome. Novais et al. [38] studied the impact of Cadmium and Zinc on *E. albidus* and also included a 4 day EC50 zinc treatment. Although this microarray study only assessed a limited amount (1124 sequences) of *E. albidus* sequences we were able to retrieve 10 orthologous transcripts that show highly comparable significant gene regulation upon Zn exposure. Two of the ten transcripts were cysteine rich proteins, which contained metallothionein-6 clusters denoting putative metallothionein genes. Other genes of interest are tropomyosin-2 and calcium binding alpha-actinin, which are both involved in the cell-skeleton integrity. Moreover, homologs of several gene families show comparable significant regulation in *E. albidus* as well as *E. crypticus* Zn exposure studies. Seven members of the ribosomal protein family are significantly up regulated in *E. crypticus*, while two ribosomal protein genes are up regulated in *E. albidus*. Also, both species show up regulation of actin, glycine N-methyltransferase and nicotinic acetylcholine receptor alpha subunit precursor homologs (Additional file 9, [39]). This indicates that substantial degree of conservation of transcriptional regulation in response to Zn stress can be observed among related species.

## Conclusions

In conclusion we showed that the current microarray platform is functional in terms of identification of transcripts associated with toxicity. Zinc-exposed animals were not included in the initial RNA pool. Still, we identified differentially expressed genes responding to zinc toxicity, which were identified in previous biochemical studies to be important in zinc detoxification. Additionally, microarray data suggest that transposon activity is altered by zinc toxicity, which are highly interesting avenues for further (functional) research. However, this requires a more elaborate experimental design that addresses developmental programming, dose–response measurements and inter-individual variation. Also. we deduce from the scientific literature that expression microarrays are nowadays more and more supplanted by RNASeq analysis. According to Robbens et al. [40] lack of standardization and cross-platform validation prevents acceptance of genomic data integration into risk assessment. However, more recently, the microarray quality control consortium provided such standardization and cross-validation data specifically for microarray platforms [41,42]. This led to acceptance of this tool in clinical and pharmaceutical testing, while RNAseq technology currently lacks such high level of standardization. It also initiated case studies that successfully integrated microarray data into environmental risk assessment [43,44], which indicates that microarray analysis will remain an important tool, especially in 21^st^ century risk assessment practice.

### Availability of supporting data

Raw sequence read data were submitted to the NCBI Sequence Read Archive under accession number [SRP024387]. The assembled *E. crypticus* contigs were submitted to the NCBI Transcriptome Shotgun Assembly (TSA) Sequence Database under the TSA accession number [GALF01000000]. Raw microarray data and its MIAME compliant metadata were deposited at NCBI Gene-Expression Omnibus under accession number [GSE51838] and platform ID [GPL17851]. All three datasets are organized under Bio-project [PRJNA207507].

## Competing interests

The authors declare that they have no competing interests.

## Authors’ contributions

MPCF: performed all exposures and RNA isolations, participated in assembly and annotation, participated in microarray construction, performed data analysis, participated in design and coordination of the study, drafted the manuscript. TEB: performed microarray construction, designed microarray experiment and performed data analysis, including microarray analysis, statistical analysis and pathway analysis, participated in drafting the manuscript. JKC: facilitated 454 pyrosequencing, assembly and annotation, participated in design and coordination of the study, helped drafting the manuscript. RV: performed microarray hybridizations and scanning. CAMG: participated in design of experimental treatments, helped planning the study, helped drafting the manuscript. NMS: helped planning the study, helped drafting the manuscript. AMVMS: helped planning the study, helped drafting the manuscript. MJBA: participated in design and coordination of the study, helped drafting the manuscript. DR: designed and coordinated the study, drafted the manuscript, participated in microarray construction, participated in annotation and data analysis. All authors read and approved the final manuscript.

## Supplementary Material

Additional file 1 Detailed description of all treatments, including data on RNA quantity and quality. Click here for file

Additional file 2 Description of compounds applied in the chemical exposure with nominal sublethal effect concentrations and CAS numbers. Click here for file

Additional file 3 Length distribution of the raw sequence reads. Click here for file

Additional file 4 **Core Eukaryotic Genes Mapping Approach (CEGMA) to assess the completeness of*E. crypticus*’ transcriptome.** The 458 core proteins from Drosophila were used for homology searches in the transcriptome. Click here for file

Additional file 5 **Summary of annotations as performed by the transcriptome annotation pipeline at CGB, Indiana University** (http://isga.cgb.indiana.edu/Pipeline/View?pipeline=248). The file is subdivided in annotations for singletons, contigs and isotigs. Click here for file

Additional file 6 **Distribution of most abundant GO terms for*E. crypticus*singletons and contigs.** Level 4 GO terms, obtained for biological processes (BP), molecular functions (MF) and cellular compartments (CC), are represented by the percentage of sequences mapped (%Seq) to singletons or contigs. Click here for file

Additional file 7 **Resulting graph of iPATH metabolic pathway explorer.** All enzyme codes from singleton & contig annotations were used as input. Blue-Grey edges represent all enzymatic reactions in iPATH explorer. Red edges represent enzymatic reactions deduced from annotation in the *E. crypticus* transcriptome. Blue-grey dots: chemical compound. Click here for file

Additional file 8 Mean Average (MA) plots for all probes (A), contigs (B) and singletons (C). Click here for file

Additional file 9 List of significantly Zinc-regulated (FDR corrected p < 0.5) transcripts with annotations. Click here for file

Additional file 10 GO acyclic graph of the significantly Zn-regulated annotated transcripts. Click here for file
