# Supplementary material for: Transcriptome assembly and microarray construction for Enchytraeus crypticus, a model oligochaete to assess stress response mechanisms derived from soil conditions
Source: BMC Genomics. 2014 Apr 23;15:302. doi: 10.1186/1471-2164-15-302 (PMC4234436; doi:10.1186/1471-2164-15-302)
Supplement: Additional file 1 — Detailed description of all treatments, including data on RNA quantity and quality. [file 1471-2164-15-302-S1.docx]

**Additional File 1**

| **Test condition** | **Replicate name** | **Sample & test description** | **Number collected** | **[RNA] (ng/μL)** | **A260 / A280** | **A260 / A230** |
| --- | --- | --- | --- | --- | --- | --- |
| Culture Control | c05 | 5 Adults directly collected from culture in agar medium. | 5 | 139.5 | 2.2 | 2.3 |
|  | c20 |  | 20 | 397.6 | 2.2 | 2.3 |
| LUFA Θ Control | WCa | Adults in LUFA Θ Control WHC 50% for 2 days (48 h, perforated, 20 ˚C) | 10 | 226.8 | 2.2 | 1.9 |
|  | WCb |  | 16 | 343.6 | 2.2 | 2.3 |
| Acetone Control | ACa | Adults at Acetone Control in LUFA WHC 50% for 2 days (48 h, perforated, 20 ˚C) | 18 | 259.8 | 2.2 | 2.3 |
|  | ACb |  | 16 | 352.6 | 2.2 | 2.3 |
| Cd EC10 2d | Cd10a | Adults at Cadmium EC10 in LUFA WHC 50% for 2 days (48 h, perforated, 20 ˚C) | 16 | 277.0 | 2.2 | 2.1 |
|  | Cd10b |  | 19 | 347.2 | 2.2 | 2.3 |
| Cd EC50 2d | Cd50a | Adults at Cadmium EC50 in LUFA WHC 50% for 2 days (48 h, perforated, 20 ˚C) | 20 | 434.2 | 2.2 | 2.3 |
|  | Cd50b |  | 19 | 322.4 | 2.2 | 2.2 |
| Cd EC80 2d | Cd80a | Adults at Cadmium EC80 in LUFA WHC 50% for 2 days (48 h, perforated, 20 ˚C) | 20 | 412.7 | 2.2 | 2.2 |
|  | Cd80b |  | 19 | 426.2 | 2.1 | 2.1 |
| Car EC10 2d | Car10a | Adults at Carbendazim EC10 in LUFA WHC 50% for 2 days (48 h, perforated, 20 ˚C) |  | 105.4 | 2.0 | 2.2 |
|  | Car10b |  |  | 30.4 | 2.0 | 1.9 |
| Car EC50 2d | Car50a | Adults at Carbendazim EC50 in LUFA WHC 50% for 2 days (48 h, perforated, 20 ˚C) |  | 136.8 | 1.9 | 2.4 |
|  | Car50b |  |  | 106.5 | 2.1 | 2.1 |
| Phe EC10 2d | Phe10a | Adults at Phe EC10 in LUFA WHC 50% for 2 days (48 h, perforated, 20 ˚C) |  | 128.1 | 1.9 | 2.3 |
|  | Phe10b |  |  | 138.4 | 2.1 | 2.3 |
| Phe EC50 2d | Phe50a | Adults at Phe EC50 in LUFA WHC 50% for 2 days (48 h, perforated, 20 ˚C) |  | 141.9 | 2.1 | 2.2 |
|  | Phe50b |  |  | 119.7 | 2.0 | 2.0 |
| PCA EC10 2d | PCA10a | Adults at PCA EC10 in LUFA WHC 50% for 2 days (48 h, perforated, 20 ˚C) | 17 | 142.5 | 1.9 | 2.4 |
|  | PCA10b |  | 16 | 117.6 | 1.8 | 2.4 |
| PCA EC50 2d | PCA50a | Adults at PCA EC50 in LUFA WHC 50% for 2 days (48 h, perforated, 20 ˚C) | 17 | 172.2 | 2.0 | 1.9 |
|  | PCA50b |  | 16 | 136.2 | 2.1 | 2.2 |
| DCA ECh 2d | DCAha | Adults at DCA hormesis in LUFA WHC 50% for 2 days (48 h, perforated, 20 ˚C) | 8 | 141.0 | 2.2 | 2.2 |
|  | DCAhb |  | 11 | 158.6 | 2.2 | 2.0 |
| DCA EC10 2d | DCA10a | Adults at DCA EC10 in LUFA WHC 50% for 2 days (48 h, perforated, 20 ˚C) | 10 | 126.5 | 2.2 | 2.2 |
|  | DCA10b |  | 19 | 329.0 | 2.2 | 1.8 |
| DCA EC50 2d | DCA50a | Adults at DCA EC50 in LUFA WHC 50% for 2 days (48 h, perforated, 20 ˚C) | 20 | 362.3 | 2.2 | 2.1 |
|  | DCA50b |  | 17 | 273.2 | 2.2 | 2.2 |
| -10 ˚C ½h | T-10.½a | Adults at -10 ˚C in LUFA WHC 50% for ½ hour (30', closed) | 13 | 271.9 | 2.2 | 2.0 |
|  | T-10.½b |  | 14 | 254.4 | 2.2 | 1.9 |
| 0 ˚C ½h | T0.½a | Adults at 0 ˚C in LUFA WHC 50% for ½ hour (30', closed) | 13 | 217.4 | 2.2 | 2.1 |
|  | T0.½b |  | 13 | 298.1 | 2.2 | 2.0 |
| 0 ˚C 1d | T0.1a | Adults at 0 ˚C in LUFA WHC 50% for 1 day (24 h, closed) | 13 | 294.7 | 2.2 | 2.1 |
|  | T0.1b |  | 12 | 219.9 | 2.2 | 2.2 |
| 0 ˚C 2d | T0.2a | Adults at 0 ˚C in LUFA WHC 50% for 2 days (48 h, closed) | 13 | 246.4 | 2.2 | 2.2 |
|  | T0.2b |  | 13 | 224.7 | 2.2 | 1.8 |
| 10 ˚C 1d | T10.1a | Adults at 10 ˚C in LUFA WHC 50% for 1 day (24 h, closed) | 19 | 284.7 | 2.2 | 2.1 |
|  | T10.1b |  | 15 | 239.5 | 2.2 | 1.8 |
| 10 ˚C 2d | T10.2a | Adults at 10 ˚C in LUFA WHC 50% for 2 days (48 h, closed) | 12 | 216.9 | 2.2 | 2.3 |
|  | T10.2b |  | 18 | 255.2 | 2.2 | 2.2 |
| 10 ˚C 4d | T10.4a | Adults at 10 ˚C in LUFA WHC 50% for 4 days (96 h, closed) | 17 | 312.4 | 2.2 | 2.3 |
|  | T10.4b |  | 18 | 211.1 | 2.2 | 2.2 |
| 20 ˚C 1d | T20.1a | Adults at 20 ˚C in LUFA WHC 50% for 1 day (24 h, closed) | 18 | 237.6 | 2.2 | 2.3 |
|  | T20.1b |  | 19 | 300.1 | 2.2 | 2.1 |
| 20 ˚C 2d | T20.2a | Adults at 20 ˚C in LUFA WHC 50% for 2 days (48 h, closed) | 19 | 453.6 | 2.2 | 2.3 |
|  | T20.2b |  | 20 | 359.3 | 2.2 | 2.3 |
| 20 ˚C 4d | T20.4a | Adults at 20 ˚C in LUFA WHC 50% for 4 days (96 h, closed) | 19 | 314.2 | 2.2 | 2.3 |
|  | T20.4b |  | 19 | 584.2 | 2.2 | 2.2 |
| 30 ˚C 1d | T30.1a | Adults at 30 ˚C in LUFA WHC 50% for 1 day (24 h, closed) | 18 | 250.8 | 2.2 | 2.3 |
|  | T30.1b |  | 20 | 246.6 | 2.2 | 2.2 |
| 30 ˚C 2d | T30.2a | Adults at 30 ˚C in LUFA WHC 50% for 2 days (48 h, closed) | 16 | 275.3 | 2.2 | 2.1 |
|  | T30.2b |  | 17 | 298.2 | 2.2 | 2.3 |
| 30 ˚C 4d | T30.4a | Adults at 30 ˚C in LUFA WHC 50% for 4 days (96 h, closed) | 18 | 161.6 | 2.2 | 1.9 |
|  | T30.4b |  | 15 | 176.7 | 2.2 | 1.8 |
| 40 ˚C HS 30' | T40HSa | Adults at 40 ˚C in LUFA WHC 50% for ½ hour (30' constant 40 ˚C, closed) | 19 | 336.0 | 2.2 | 1.9 |
|  | T40HSb |  | 19 | 219.6 | 2.2 | 2.1 |
| 40 ˚C HS 30' | T40HSc | Adults at 40 ˚C in LUFA WHC 50% for ½ hour (30' constant 40 ˚C, closed) |  | 366.4 | 2.2 | 2.3 |
|  | T40HSd |  |  | 414.4 | 2.2 | 2.3 |
| 20% WHC 1d | 20%1a | Adults in LUFA WHC 20% for 1 day (24 h, closed, 20 ˚C) | 15 | 231.7 | 2.2 | 2.1 |
|  | 20%1b |  | 14 | 297.1 | 2.2 | 2.2 |
| 20% WHC 2d | 20%2a | Adults in LUFA WHC 20% for 2 days | 10 | 160.2 | 2.2 | 2.2 |
|  | 20%2b |  | 8 | 170.9 | 2.2 | 2.3 |
| 20% WHC 4d | 20%4a | Adults in LUFA WHC 20% for 4 days | 3 | 11.8 | 2.2 | 1.8 |
| 50% WHC 2d | 50%2a | Adults in LUFA WHC 50% for 2 days | 10 | 239.2 | 2.2 | 2.3 |
|  | 50%2b |  | 10 | 214.5 | 2.2 | 2.2 |
| 50% WHC 4d | 50%4a | Adults in LUFA WHC 50% for 4 days | 9 | 146.5 | 2.2 | 2.3 |
|  | 50%4b |  | 10 | 180.3 | 2.2 | 2.3 |
| 90% WHC 2d | 90%2a | Adults in LUFA WHC 90% for 2 days | 9 | 124.2 | 2.2 | 2.3 |
|  | 90%2b |  | 10 | 182.9 | 2.2 | 2.3 |
| 90% WHC 4d | 90%4a | Adults in LUFA WHC 90% for 4 days | 9 | 127.8 | 2.2 | 2.2 |
|  | 90%4b |  | 10 | 209.0 | 2.2 | 2.3 |
| pH=3 2d | pH3a | Adults at pH=3 in OECD WHC 50% for 2 days (48 h, perforated, 20 ˚C) | 11 | 404.4 | 2.2 | 2.4 |
|  | pH3b |  | 16 | 248.1 | 2.2 | 2.2 |
| pH=4,5 2d | pH4,5a | Adults at pH=4,5 in OECD WHC 50% for 2 days (48 h, perforated, 20 ˚C) | 16 | 219.7 | 2.2 | 2.2 |
|  | pH4,5b |  | 16 | 195.1 | 2.2 | 2.0 |
| pH=6 2d | pH6a | Adults at pH=6 in OECD WHC 50% for 2 days (48 h, perforated, 20 ˚C) | 12 | 145.7 | 2.2 | 2.2 |
|  | pH6b |  | 14 | 174.7 | 2.2 | 2.3 |
| Cocoons | Csa | Cocoons (white/light yellow) collected from cultures at 16 ˚C | 20 | 55.6 | 1.9 | 2.4 |
|  | 30coc |  | 30 | 117.9 | 1.8 | 2.4 |
| Synchronized Fresh Cocoons | FCS56 | Cocoons collected from synchronized cultures at 16 ˚C (24h & 6h respectively) | 34 | 82.3 | 1.9 | 2.4 |
|  | 628FC6h |  | 53 | 41.6 | 2.2 | 2.0 |
| Juveniles | Juv.a | Synchronized juveniles (20 ˚C) |  | 157.1 | 2.2 | 2.2 |
|  | Juv.b |  |  | 197.1 | 2.2 | 2.2 |
| Fragmentation & Regeneration | FR.a | Fragmenting adults and fragments (including minor fragments) |  | 42.0 | 2.1 | 1.8 |
|  | FR.b |  |  | 318.6 | 2.2 | 2.2 |
| Starvation (agar) | ☼Sa | Adults in medium without food (20 ˚C) |  | 150.9 | 2.2 | 2.3 |
|  | ☼Sb |  |  | 133.5 | 2.2 | 2.3 |
| Deprivation | Dep˚ | Adults without food, water or air (20 ˚C) | 10 | 187.1 | 2.2 | 2.3 |
