# Supplementary material for: Transcriptome assembly and microarray construction for Enchytraeus crypticus, a model oligochaete to assess stress response mechanisms derived from soil conditions
Source: BMC Genomics. 2014 Apr 23;15:302. doi: 10.1186/1471-2164-15-302 (PMC4234436; doi:10.1186/1471-2164-15-302)
Supplement: Additional file 2 — Description of compounds applied in the chemical exposure with nominal sublethal effect concentrations and CAS numbers. [file 1471-2164-15-302-S2.docx]

**Table S2**

| **Test chemical** | **Cadmium chloride hemi-pentahydrate** | | **Carbendazim** | | **Phenanthrene** | | **Penta-ChloroAniline** | | **3,5-Di-ChloroAniline** | |
| --- | --- | --- | --- | --- | --- | --- | --- | --- | --- | --- |
| **Chemical formula** | CdCl_2·_2½H_2_O | | C_9_H_9_N_3_O_2_ | | C_14_H_10_ | | C_6_H_2_Cl_5_N | | C_6_H_5_Cl_2_N | |
| **CAS number** | 7790-78-5 | | 10605-21-7 | | 85-01-8 | | 527-20-8 | | 626-43-7 | |
| **Nominal concentration (mg kg^−1^)** | EC10 | 15 | EC10 | 3.40E-06 | EC10 | 37 | EC10 | 3 | EC10 | 60 |
|  | EC50 | 35 | EC50 | 4.50E-03 | EC50 | 145 | EC50 | 275 | EC50 | 102 |
|  | EC80 |  |  | | | | | | Ech | 50 |
