# Supplementary figures and images for: Transcriptome assembly and microarray construction for Enchytraeus crypticus, a model oligochaete to assess stress response mechanisms derived from soil conditions
Source: BMC Genomics. 2014 Apr 23;15:302. doi: 10.1186/1471-2164-15-302 (PMC4234436; doi:10.1186/1471-2164-15-302)

**Additional File 3**


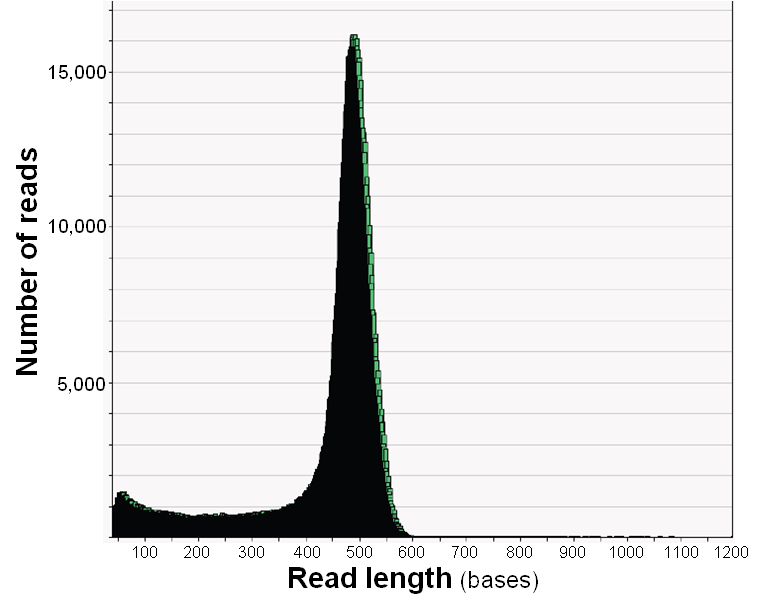

Supplement: Additional file 3 — Length distribution of the raw sequence reads. [file 1471-2164-15-302-S3.docx]

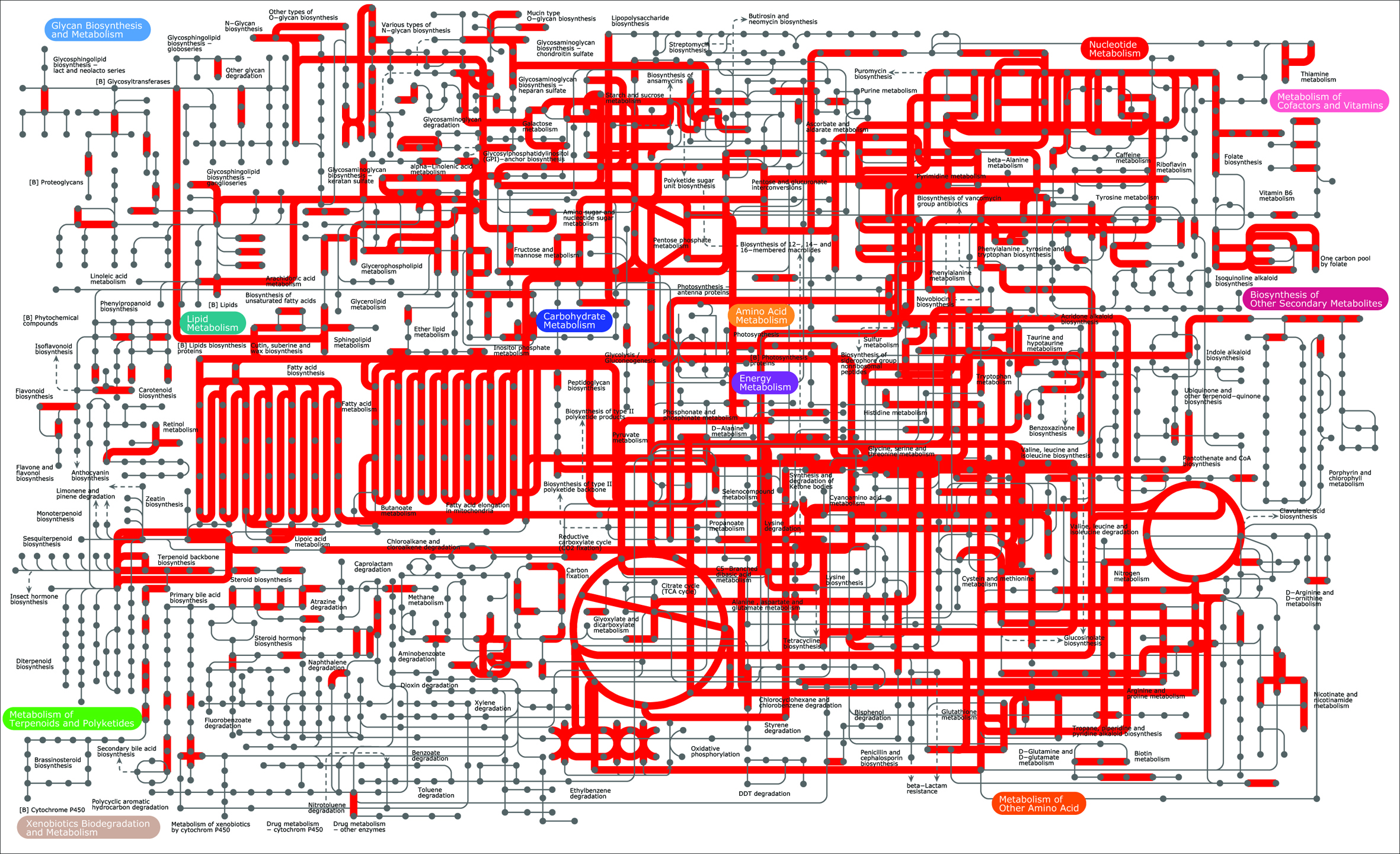

Supplement: Additional file 7 — Resulting graph of iPATH metabolic pathway explorer. All enzyme codes from singleton & contig annotations were used as input. Blue-Grey edges represent all enzymatic reactions in iPATH explorer. Red edges represent enzymatic reactions deduced from annotation in the E. crypticus transcriptome. Blue-grey dots: chemical compound. [file 1471-2164-15-302-S7.jpeg]

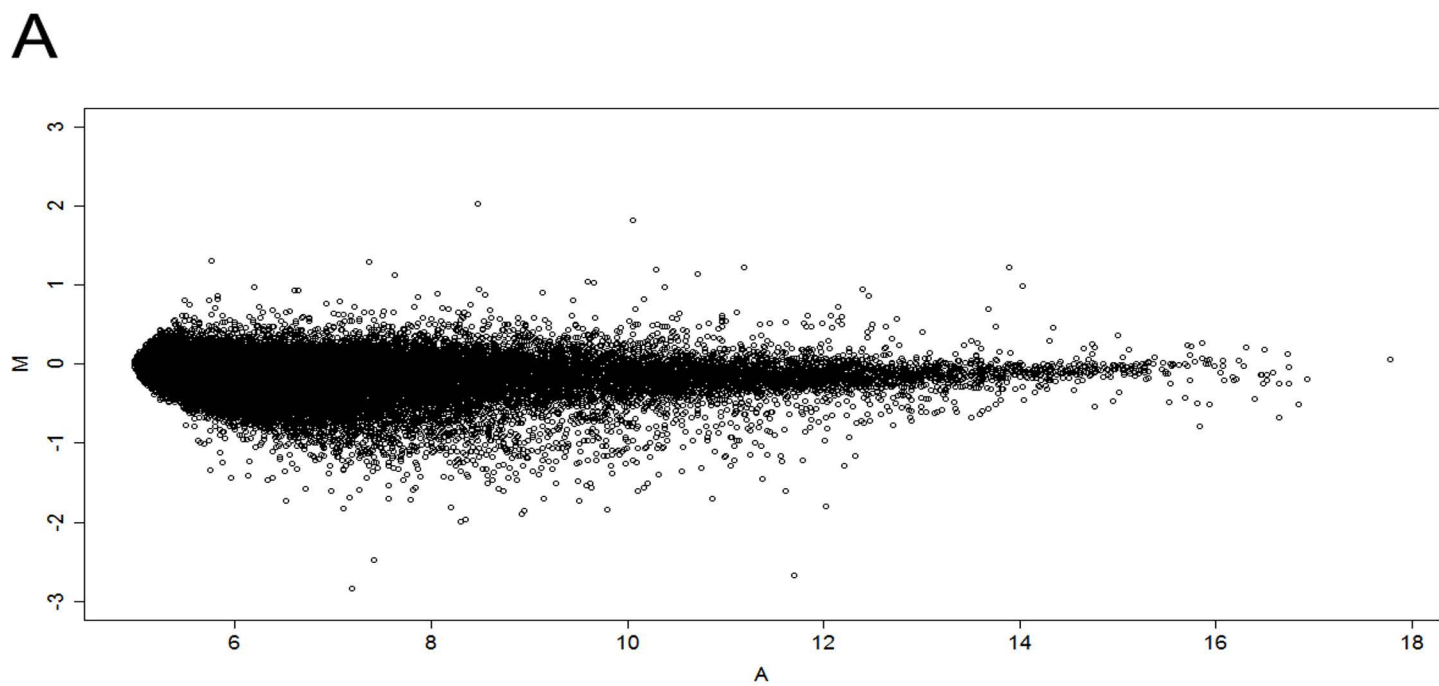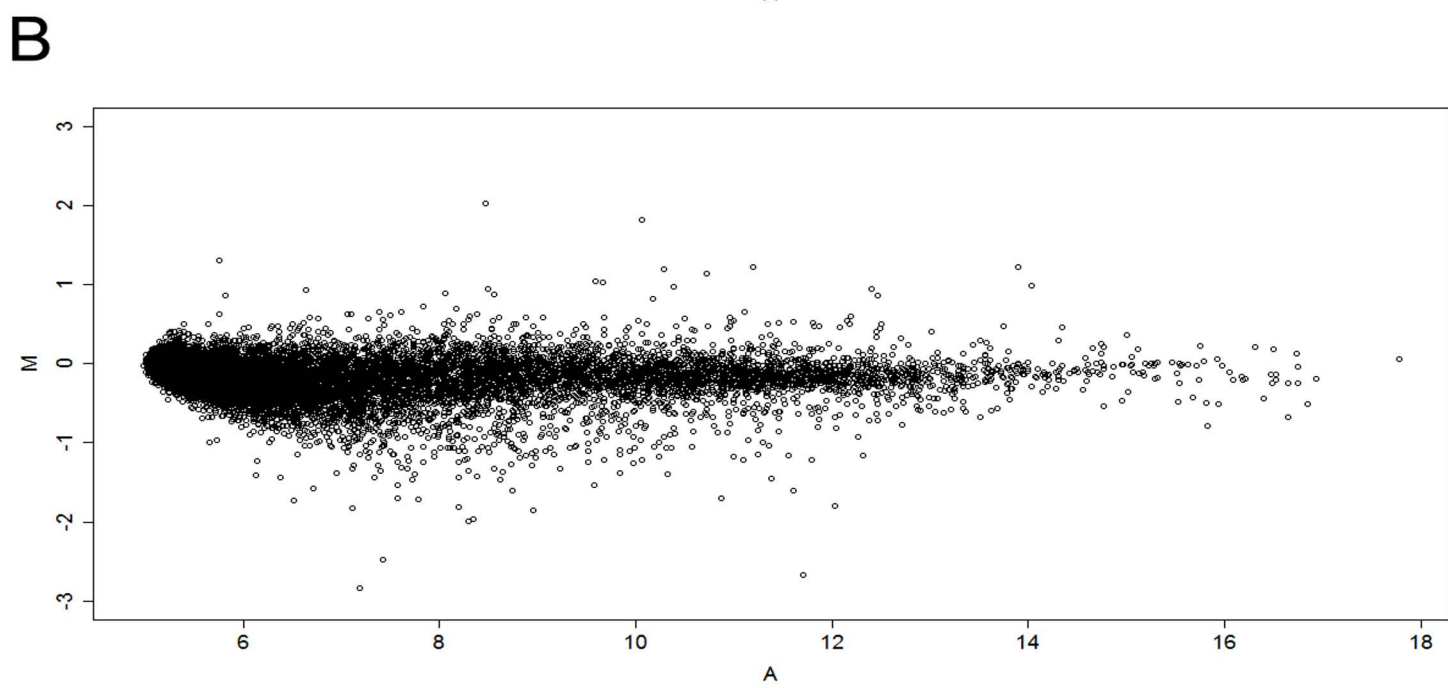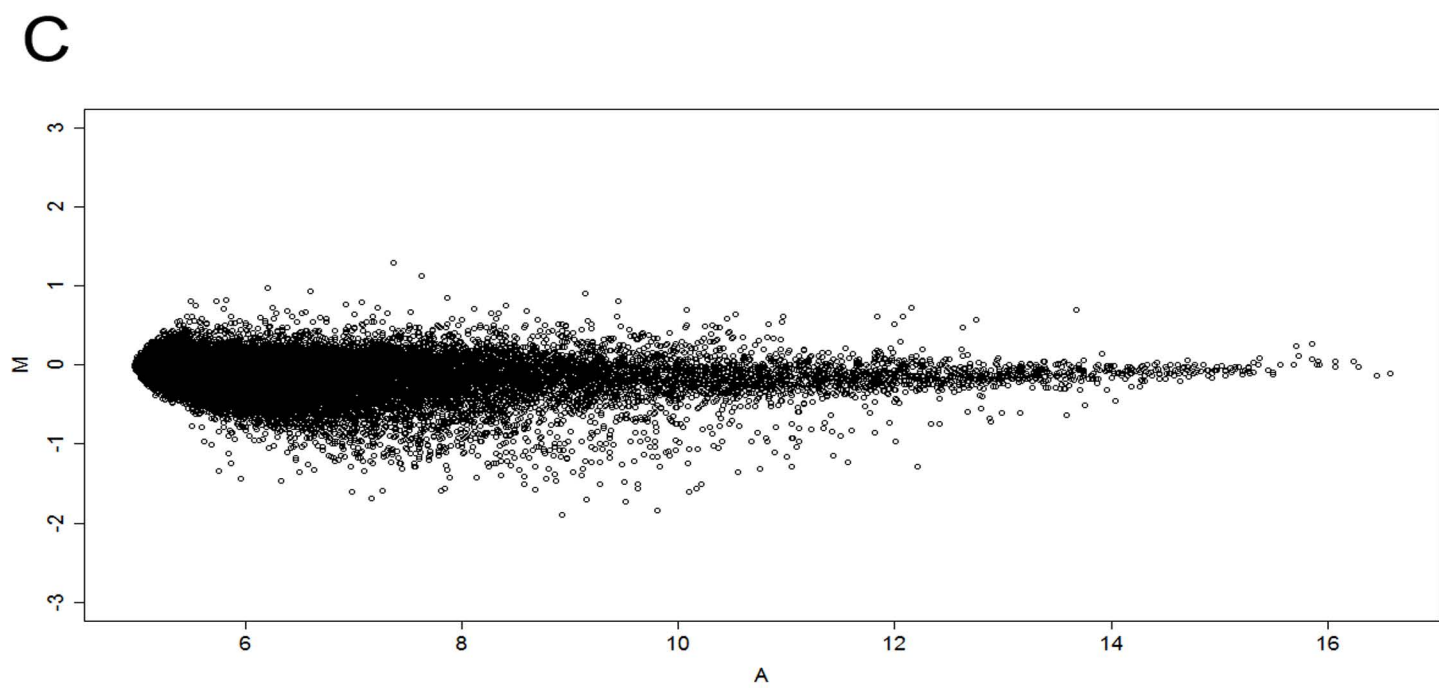

Supplement: Additional file 8 — Mean Average (MA) plots for all probes (A), contigs (B) and singletons (C). [file 1471-2164-15-302-S8.pdf]

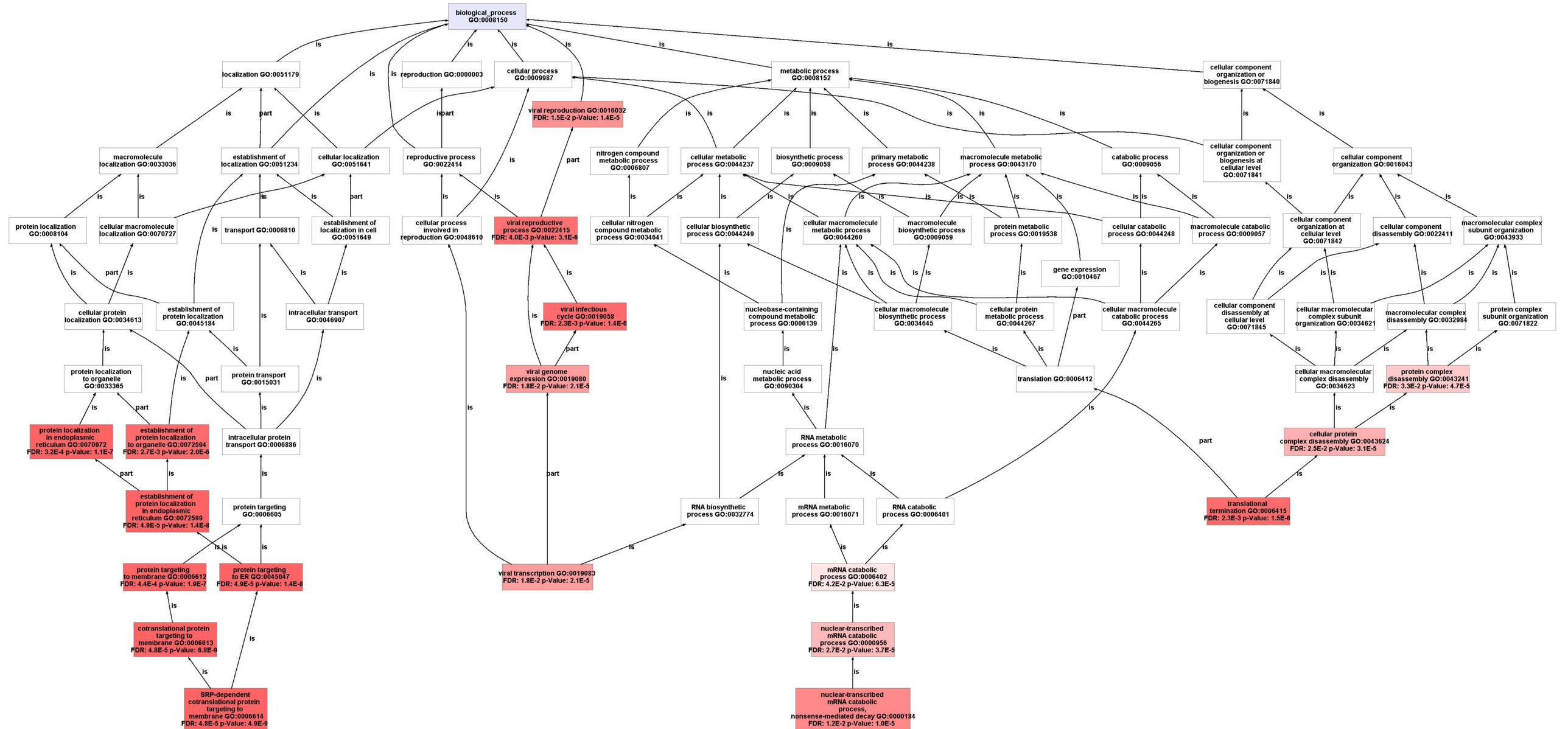

Enriched Graph

Supplement: Additional file 10 — GO acyclic graph of the significantly Zn-regulated annotated transcripts. [file 1471-2164-15-302-S10.pdf]
