# Supplementary material for: Transcriptome assembly and microarray construction for Enchytraeus crypticus, a model oligochaete to assess stress response mechanisms derived from soil conditions
Source: BMC Genomics. 2014 Apr 23;15:302. doi: 10.1186/1471-2164-15-302 (PMC4234436; doi:10.1186/1471-2164-15-302)
Supplement: Additional file 6 — Distribution of most abundant GO terms forE. crypticussingletons and contigs. Level 4 GO terms, obtained for biological processes (BP), molecular functions (MF) and cellular compartments (CC), are represented by the percentage of sequences mapped (%Seq) to singletons or contigs. [file 1471-2164-15-302-S6.docx]

### Figure 4: Distribution of most abundant GO terms for *E. crypticus* singletons and contigs. Level 4 GO terms, obtained for biological processes (BP), molecular functions (MF) and cellular compartments (CC), are represented by the percentage of sequences mapped (%Seq) of singletons and contigs.

**%Seq**

**%Seq**

**BP**

**CC**

**MF**

**BP**

**MF**

**CC**
